# Data-Driven Fault Diagnosis Techniques: Non-Linear Directional Residual vs. Machine-Learning-Based Methods

**DOI:** 10.3390/s22072635

**Published:** 2022-03-29

**Authors:** Nicholas Cartocci, Marcello R. Napolitano, Francesco Crocetti, Gabriele Costante, Paolo Valigi, Mario L. Fravolini

**Affiliations:** 1 Department of Engineering, University of Perugia, Via G. Duranti 67, 06125 Perugia, Italy; nicholas.cartocci@unipg.it (N.C.); francesco.crocetti@unipg.it (F.C.); gabriele.costante@unipg.it (G.C.); paolo.valigi@unipg.it (P.V.); 2 Department of Mechanical and Aerospace Engineering, West Virginia University, Morgantown, WV 26506, USA; marcello.napolitano@mail.wvu.edu

**Keywords:** additive model, anomaly detection, multivariate adaptive regression splines, time-dependent directional residuals, non-linear residual-based technique, fault isolation, fault estimation

## Abstract

Linear dependence of variables is a commonly used assumption in most diagnostic systems for which many robust methodologies have been developed over the years. In case the system nonlinearities are relevant, fault diagnosis methods, relying on the assumption of linearity, might potentially provide unsatisfactory results in terms of false alarms and missed detections. In recent years, many authors have proposed machine learning (ML) techniques to improve fault diagnosis performance to mitigate this problem. Although very powerful, these techniques require faulty data samples that are representative of any fault scenario. Additionally, ML techniques suffer from issues related to overfitting and unpredictable performance in regions which are not fully explored in the training phase. This paper proposes a non-linear additive model to characterize the non-linear redundancy relationships among the system signals. Using the multivariate adaptive regression splines (MARS) algorithm, these relationships are identified directly from the data. Next, the non-linear redundancy relationships are linearized to derive a local time-dependent fault signature matrix. The faulty sensor can then be isolated by measuring the angular distance between the column vectors of the fault signature matrix and the primary residual vector. A quantitative analysis of fault isolation and fault estimation performance is performed by exploiting real data from multiple flights of a semi-autonomous aircraft, thus allowing a detailed quantitative comparison with state-of-the-art machine-learning-based fault diagnosis algorithms.

## 1. Introduction

Fault diagnosis (FDi) systems are essential components of many engineering systems. These systems play a very important role for accident prevention, service continuity, and cost minimization, ultimately leading to increased human safety in transportation systems. Due to the increasing availability of multi-source sensorial data along with the significant computational power and storage capacity of modern computers, today, there is an increasing research interest in developing data-driven (DD) algorithms to tackle complex monitoring and control problems [1]. DD approaches are widely used in applications where a detailed physical knowledge of the system is unavailable or not readily available or in cases where system input-output relations are too complex or too uncertain. Typically, DD approaches applied to FD problems derive fault-sensitive signals (also known as diagnostic signals) directly from experimental models identified from sample datasets acquired from the monitored system during normal and faulty operations. The pioneers in this field are Isermann [2,3,4], Basseville [5,6,7], and Gertler [8,9,10].

Today, multivariate statistical process monitoring (SPM) methods, such as principal component analysis (PCA) [11] (and its variants) and parity space approaches [12,13,14,15], are widespread techniques used for system monitoring and fault diagnosis purposes. The widespread use of PCA-based monitoring techniques is due to its simplicity and its capability to efficiently manage large quantities of multivariate data. In this context, widespread approaches used for fault isolation (FI) are the so-called contribution plots methods, such as the reconstruction-based contributions method [11,13]. Although powerful and effective, this method might produce incorrect fault isolations due to the so-called smearing effect, i.e., the influence of a faulty sensor measurement on non-faulty sensors contributions. In a system characterized by a limited number of monitored variables, directional residuals methods have shown to be a valid alternative to SPM methods. Directional residual methods work by relating possible faulty sensors with the characteristic direction of the faults, isolating the faulty sensor as the one having the smallest distance between the residuals signal and the monitored sensor fault directions [12,13,16]. An important variant of the directional residual method is the structured residual approach [9].

A fundamental assumption underlying most SPM techniques is the linear dependence between modeling variables. While this assumption may be reasonable in some applications, in the case of strongly non-linear systems or significant variations in the operating range of the signals, these techniques could lead to inaccurate results. The direct consequence on fault diagnosis is that the linearity in covariate FD models may produce many false positives and false negatives in practical applications when applied to non-linear systems.

A typical approach to allow non-linear dependencies between the covariates is to augment their number by introducing several new terms derived by non-linear transformations of the original signals while retaining the linear dependence between the extended set of covariates. Although this approach is straightforward, it suffers from the problem that the set of extended non-linear functions cannot be defined ‘a priori’. Instead, a complex and time-consuming tuning phase is needed to select a suitable set of non-linear transformations. These problems have motivated the scientific community to go beyond the linear dependence assumptions of covariates, promoting the development of non-parametric models based on generic non-linear smooth functions, such as splines, neural networks, and kernel models, and implement specific data-driven tuning algorithms to reduce the mean and variance of the mapping error to identify best-fitting models [17].

Another direction to deal with the problem of fault diagnosis in non-linear systems is related to the use of machine learning and deep learning techniques. The issue of Fault Isolation, in fact, can be easily set as a direct application of a classification or clustering problem. At the same time, ML regression techniques can also be employed for estimating the shape and amplitude of the fault (Fault Estimation (FE)) [18,19,20,21].

An interesting comparison of FI and FE performance of three popular classification algorithms, namely the support vector machine (SVM), K-nearest neighbor (KNN), and decision tree (DT), can be found in [17]. The classification algorithms are trained using data from optimized and non-optimized sensor subsets and then validated with new data characterized by varying degrees of fault severity. In [22], Erfani et al. present a hybrid model in which an unsupervised deep belief network (DBN) is trained to extract latent features. Then, a one-class support vector machine (SVM) based on the DBN features is trained to learn decision surfaces. In [23], Revathi and Kumar proposed a deep-learning-based anomaly detection and classification system in video sequences, where the final module classifies the detected events as usual or suspect. In [24], Pashazadeh et al. propose a data-driven fault detection and isolation (FDI) scheme based on the fusion of different classifiers for a wind turbine challenge system. Multi-layer perceptron (MLP), radial basis function (RBF), decision tree (DT), and K-nearest neighbor (KNN) classifiers are implemented in parallel, and the faulty sensor is identified using a majority voting method. Next, discrete-time up–down counters (UDCs) are used for each fault to reduce false alarms (FAs) and missed detections (MDs). In [25], an efficient strategy for fault detection and isolation (FDI) for an industrial gas turbine based on ensemble learning methods is introduced; specifically, a fault isolation scheme based on ensemble bagged trees is developed to isolate faults in a steady-state runtime.

In this study, we considered a natural non-linear extension of linear regression models of the form $\sum_{1}^{p}w_{i}x_{i}$, i.e., the class of the so-called generalized additive models (GAM) [26] $\sum_{1}^{p}w_{i}g_{i}(x_{i})$, where $g_{i}(x_{i})$ are generic smooth functions to be identified from data. Due to the simple additive structure, GAMs are sufficiently versatile for capturing linear or non-linear relationships between response functions and covariates. The additive form is of particular interest for FDi problems because it allows the easy calculation of the fault sensitivity of the individual monitored variables. Although GAMs have been used extensively in many application fields, their employment in a data-driven FDi system has not been fully explored. In fact, to date, only a few studies have been presented, such as [16,27]. Motivated by the mentioned issues, in this paper, we propose GAMs to identify non-linear parity relationships in the monitored variables using cubic spline basis functions to characterize the non-linear functions $g_{i}(x_{i})$ by exploiting the MARS modeling and estimation algorithm proposed in [28].

Next, to exploit the consolidated tools available for the fault diagnosis of linear parity space models, a local linearization of the identified GAM parity relations is performed to achieve a time-dependent fault sensitivity matrix.

Indeed, unlike standard linear parity methods, the so-called fault signature matrix is not constant but, rather, it depends on the operating point, implying that the resulting fault directions are not constant but time-varying. A state-dependent fault sensitivity model can better capture the effects of faults in a non-linear system compared to a standard linear and fixed fault sensitivity matrix. The proposed GAM plus linearization approach can immediately fit the directional residual FI method developed in the linear contest and applied in [14,15]. In addition to this first key innovative aspect, this study aims to show the effectiveness of the proposed technique compared to machine learning techniques applied to the problems of fault isolation and fault estimation. In particular, the second main contribution of this research is related to the comparison of the proposed directional residual-based technique with ML fault diagnosis methods. This is performed by investigating the advantages and disadvantages of each approach, highlighting the benefits in terms of performance, memory occupancy and robustness to unexpected fault amplitudes.

The proposed novel method is applied to design a complete fault isolation and estimation (FIE) system based on real sensor data taken from a semi-autonomous aircraft where single additive faults are artificially injected on eight primary sensors.

## 2. Non-Linear Additive Models for Fault Diagnosis

The set of the monitored (potentially faulty) sensors measurements is concatenated in the vector $x(k)\in {\mathbb{R}}^{n_{x}}$, while the set of control signals and other no monitored sensors (assumed not faulty) are included in the vector $u(k)\in {\mathbb{R}}^{n_{u}}$. The integer k is the discrete-time index at sample time t=k⋅Δt (where Δt is the sampling interval). Occasionally in the article, the dependence on k is omitted to simplify the notation. The proposed FD technique is based on analytical redundancy (AR) concepts [12,13,16]. It is assumed that a sensor measurement $x_{i}(k)$ is approximated by a non-linear additive model consisting of the linear combination of non-linear functions ($g_{i,j}$ and $h_{i,j}$) defined as follows

(1) $${\hat{x}}_{i}(k)=\sum_{\underset{j\neq i}{j=1}}^{n_{x}}w_{xi,j}g_{i,j}(x_{j})+\sum_{j=1}^{n_{u}+1}w_{ui,j}h_{i,j}(u_{j})\;i=1\ldots n_{x}$$

where $w_{xi,j}$ and $w_{ui,j}$ are constant coefficients (to be estimated from data) and $g_{i,j}$ and $h_{i,j}$ are non-linear functions of the variables $x_{j}(k)$ and $u_{j}(k)$, respectively, typically representing a rectified linear unit (ReLU) or Gaussians or polynomial splines [26]. The functions $h_{i,n_{u}+1}$ are assumed to be constant and equal to one in order to take into account possible constant offsets in the models. The actual signal $x_{i}(k)$ is

(2) $$x_{i}(k)={\hat{x}}_{i}(k)+\Delta_{i}(k)\;i=1\ldots n_{x}$$

where $\Delta_{i}(k)$ characterizes the modeling error and sensor noise associated with the i-th sensor. The primary residual associated to each sensor is defined as

(3) $$r_{i}(k)={\hat{x}}_{i}(k)-x_{i}(k)\;i=1\ldots n_{x}$$

At fault-free conditions, it results in the following:

(4) $$r_{i}(k)=-\Delta_{i}(k)\;i=1\ldots n_{x}$$

i.e., the residual is equal to the modeling uncertainty. Typically, $\left|\Delta_{i}(k)\right|$ is a small amplitude signal, and in the ideal perfect modeling noise-free case, this is equal to 0. In the present study, we considered the occurrence of single additive sensor faults $f_{j}(k)\in R$ on the generic j-th sensor. In the presence of a sensor fault, the fault-free measurement $x_{j}(k)$ should be substituted by the faulty signal $x_{j}(k)+f_{j}(k)\to x_{j}(k)$, that is

(5) $$x_{j}(k)+f_{j}(k)\to x_{j}(k)$$

where $f_{j}(k)$ is a generic fault modelling function that is zero in fault-free conditions and different from zero in the presence of the sensor fault. When a fault is present on the j-th sensor, the impact on the residuals can be evaluated by substituting (5) in (1) and (3). It is immediate to verify that

(6) $$\begin{array}{l} r_{i}(k)=w_{xi,j}[g_{i,j}(x_{j}(k)+f_{j}(k))-g_{i,j}(x_{j}(k))]+\Delta_{i}(k)\;i\neq j\\ r_{i}(k)=-f_{i}(k)+\Delta_{i}(k)\;i=j \end{array}$$

In the above Equation (6), the term $g_{i,j}(x_{j}(k))$ is not directly computable because the sensor reading is equal to $x_{j}(k)+f_{j}(k)$ and not to $x_{j}(k)$ in the presence of a fault. For this reason, the Taylor approximation of $g_{i,j}(x_{j}+f_{j})$ around $x_{j}+f_{j}$ is computed, that is

(7) $$g_{i,j}(x_{j}+f_{j}+\delta f_{j})=g_{i,j}(x_{j}+f_{j})+{\left.\frac{\partial g_{i,j}(x_{j}+f_{j})}{\partial f_{j}}\right|}_{x_{j}+f_{j}}\delta f_{j}+\Delta g_{ij}$$

where $\delta f_{j}$ is a fault increment and $\Delta g_{ij}$ contains higher-order terms of the Taylor expansion. In this study, we exploited model (7) to compute an approximation of $g_{i,j}(x_{j})$. This can be easily achieved by taking $\delta f_{j}=-f_{j}$ in (7) (in fact, results $g_{i,j}(x_{j}+f_{j}+\delta f_{j})=g_{i,j}(x_{j}+f_{j}-f_{j})=g_{i,j}(x_{j})$) resulting in:

(8) $$g_{i,j}(x_{j})=g_{i,j}(x_{j}+f_{j})+{\left.\frac{\partial g_{i,j}(x_{j}+f_{j})}{\partial f_{j}}\right|}_{x_{j}+f_{j}}(-f_{j})+\Delta g_{ij}$$

Substituting Expression (8) for $g_{i,j}(x_{j})$ in the residuals in (6) results in the following:

(9) $$r_{i}(k)=w_{xi,j}[g_{i,j}(x_{j}+f_{j})-g_{i,j}(x_{j}+f_{j})-{\left.\frac{\partial g_{i,j}(x_{j}+f_{j})}{\partial f_{j}}\right|}_{x_{j}+f_{j}}(-f_{j})-\Delta g_{ij}]+\Delta_{i}\;i\neq j$$

that is

(10) $$r_{i}(k)=w_{xi,j}{\left.\frac{\partial g_{i,j}(x_{j}+f_{j})}{\partial f_{j}}\right|}_{x_{j}+f_{j}}f_{j}+[\Delta_{i}-w_{xi,j}\Delta g_{ij}]\;i\neq j$$

Define:

(11) $${\bar{w}}_{i,j}(x_{j}+f_{j})=w_{xi,j}{\left.\frac{\partial g_{i,j}(x_{j}+f_{j})}{\partial f_{j}}\right|}_{x_{j}+f_{j}}i\neq j$$

then (10) becomes

(12) $$r_{i}(k)={\bar{w}}_{i,j}(x_{j}+f_{j})f_{j}+[\Delta_{i}-w_{i,j}\Delta g_{ij}]\;i\neq j$$

the above expressions can be arranged in matrix form resulting in:

(13) $$\left[\begin{array}{c} r_{1}(k)\\ r_{2}(k)\\ r_{3}(k)\\ \ldots \\ r_{n_{x}}(k) \end{array}\right]=\left[\begin{array}{ccccc} -1 & {\bar{w}}_{1,2}(x_{2}+f_{2}) & {\bar{w}}_{1,3}(x_{3}+f_{3}) & \ldots & {\bar{w}}_{1,nx}(x_{nx}+f_{nx})\\ {\bar{w}}_{2,1}(x_{1}+f_{1}) & -1 & {\bar{w}}_{2,3}(x_{3}+f_{3}) & \ldots & {\bar{w}}_{2,nx}(x_{nx}+f_{nx})\\ {\bar{w}}_{3,1}(x_{1}+f_{1}) & {\bar{w}}_{3,2}(x_{2}+f_{2}) & -1 & \ldots & {\bar{w}}_{3,nx}(x_{nx}+f_{nx})\\ \ldots & \ldots & \ldots & \ldots & \ldots \\ {\bar{w}}_{nx,1}(x_{1}+f_{1}) & {\bar{w}}_{nx,2}(x_{2}+f_{2}) & {\bar{w}}_{nx,3}(x_{3}+f_{3}) & \ldots & -1 \end{array}\right]\left[\begin{array}{c} f_{1}\\ f_{2}\\ f_{3}\\ \ldots \\ f_{nx} \end{array}\right]+\bar{\Delta }$$

The matrix in (13) ($\bar{W}(k)\in {\mathbb{R}}^{n_{x}\times n_{x}}$) is known as the fault sensitivity matrix and $\bar{\Delta }\in {\mathbb{R}}^{n_{x}\times n_{x}}$ contains all the uncertain terms in (13). It is observed that the matrix $\bar{W}(k)$ is time-dependent; in other words, it depends on the current measurements at time *k*.

The occurrence of a single fault at a time on a generic j-th sensor is assumed here; therefore, in (13), only the component $f_{j}$ is different from zero (that is ${\left[\begin{array}{ccccc} f_{1} & f_{2} & f_{3} & \ldots & f_{nx} \end{array}\right]}^{T}={\left[\begin{array}{ccccc} 0 & \ldots & f_{j} & \ldots & 0 \end{array}\right]}^{T}$). This implies that (13) simplifies to

(14) $$\left[\begin{array}{c} r_{1}(k)\\ r_{2}(k)\\ \ldots \\ \ldots \\ r_{n_{x}}(k) \end{array}\right]=\left[\begin{array}{c} {\bar{w}}_{1,j}(x_{j}+f_{j})\\ \ldots \\ -1\\ \ldots \\ {\bar{w}}_{nx,j}(x_{j}+f_{j}) \end{array}\right]f_{j}+{\bar{\Delta }}_{j}$$

In vector form

(15) $$r(k)={\bar{w}}_{j}(k)f_{j}(k)+{\bar{\Delta }}_{j}\;j=1\ldots n_{x}$$

where $r(k)\in {\mathbb{R}}^{n_{x}}$ is the primary residual vector and ${\bar{w}}_{j}(k)\in {\mathbb{R}}^{n_{x}}$ (the j-th column vectors of the matrix $\bar{W}(k)$) defines the so-called fault direction. Assuming a sufficiently large fault $f_{j}$ compared to ${\bar{\Delta }}_{j}$, the residual vector direction tends to be alienated to the known direction of the vector ${\bar{w}}_{j}(k)$. This directional information will be later exploited for sensor FI purposes. Unlike our previous papers in [12,13]—where the faults signature matrix is constant by construction—the fault signature matrix is time-varying in the present study.

### 2.1. Linear Model Case

In case the functions $g_{i,j}$ and $h_{i,j}$ are approximated by simple linear in the variables models, then Equation (1) simplifies to:

(16) $${\hat{x}}_{i}(k)=\sum_{\underset{j\neq i}{j=1}}^{n_{x}}w_{xi,j}x_{j}+\sum_{j=1}^{n_{u}+1}w_{ui,j}u_{j}\;i=1\ldots n_{x}$$

where $w_{xi,j}$ and $w_{ui,j}$ are constant weights, implying that the time-dependent matrix $\bar{W}(k)$ becomes a constant matrix W [12,13] and the associated fault directions ${\bar{w}}_{j}\;j=1\ldots n_{x}$ are fixed and constant vectors.

## 3. Fault Diagnosis (FDi)

### 3.1. Fault Isolation (FI)

Starting from the considerations in Section 2 and considering Equation (15), the faulty sensor is inferred (isolated) by exploiting the fault directional properties of matrix $\bar{W}(k)$. Specifically, the faulty sensor is isolated by evaluating the angular distances between the direction of the residual vector r(k) and the $n_{x}$ fault directions, i.e., the columns of the matrix $\bar{W}(k)$. The sensor fault direction with the lowest angular distance from the residual direction flags the faulty sensor, that is

(17) $$I_{F}(k)⇐\underset{j\in 1,\ldots ,n_{x}}{argmin}∠(r(k),\bar{W}(k))$$

where $I_{F}(k)$ is the fault index function that takes values from 1 to $n_{x}$ and indicates, at time k, the index of the isolated faulty sensor. This FI technique is previously applied in [12,13,14,15]. The same FI method is also unchanged when the simple linear models in (16) are used, with the only difference that the fault directions are now constant in time because, in this case, the matrix W is constant.

### 3.2. Fault Estimation (FE)

An advantage of operating with primary residuals, as defined in (3), is that the fault amplitude can be estimated directly. In fact, starting from Equation (15), assuming a negligible effect of Δ(k), the fault amplitude can be directly calculated as

(18) $$f_{j}(k)={\left[{\bar{w}}_{j}(k)\right]}^{-1}r(k)$$

The same FE method also applies in the case of the linear models (16).

NOTE-1: Before the FI and FE phases there is usually a fault detection (FD) phase dedicated to the detection of the occurrence of a generic anomaly condition. Since the primary purpose of this study is focused on the evaluation of FI and FE algorithms, we assumed here an “ideal” fault FD, i.e., the additive fault is detected as soon as it is injected into the sensors. Clearly, in practice, an FD delay and missed detection might be possible. The issue of FD is addressed in many studies; a detailed overview of FD techniques can be found in [29,30]. In addition, the authors have also discussed the issue of data-driven FD in previous works, e.g., in [12,14,15].

## 4. Multivariate Adaptive Regression Splines (MARS)

In this study, the data-driven identification of the non-linear functions ($g_{i,j}$ and $h_{i,j}$) defined in (1) is performed using Friedman’s multivariate adaptive regression Splines algorithm [28] which is a well-known procedure used to identify non-parametric additive models from data. The MARS algorithm can be easily set to fit the structure of the non-linear additive models in (1). In practice, the identification of the primary residuals is performed by exploiting the adaptive regression splines toolbox [31] (ARESLab). MARS is a non-parametric regression technique and can be viewed as a non-linear extension of linear regression models that can be used to model non-linear dependencies in high-dimensional data. Technically, MARS models consist of the linear combination of spline basis functions; in ARESLab, the number of basis functions and the parameters characterizing their shape is inferred directly from data through a forward–backward iterative approach [28,31]. Starting from (1), the considered MARS functions have the following form:

(19) $$\begin{array}{l} g_{i,j}(x_{j})=\sum_{m_{x}=1}^{M_{x,i,j}}\alpha_{i,j,m_{x}}B_{i,j,m_{x}}(x_{j})\\ h_{i,j}(u_{j})=\sum_{m_{u}=1}^{M_{u,i,j}}\beta_{i,j,m_{u}}B_{i,j,m_{u}}(u_{j}) \end{array}$$

where $M_{x,i,j}$ is the number of basis functions that are selected by the MARS forward–backward iterative approach to identify the $g_{i,j}$ function. The $\alpha_{i,j,m_{x}}$ are constant coefficients and $B_{i,j,m_{x}}$ is the $m_{x}$-th basis function that depends only on the $x_{j}$ variable. Similar definitions can be attributed to $M_{u,i,j}$, $\beta_{i,j,m_{u}}$, and $B_{i,j,m_{u}}$.

The ARESLab allows the selection of different classes of basis functions, such as piecewise ReLU and piecewise cubic splines. We used piecewise continuous cubic splines with continuous first derivatives to estimate the non-linear functions. An in-depth discussion and comparison between piecewise cubic models and piecewise linear models can be found in [28].

The considered piecewise cubic spline basis functions consist of one or two “complementary” basis functions $B(x|s,\kappa_{-},\kappa ,\kappa_{+})$. These are defined as follows:

(20) $$B(x|s=1,\kappa_{-},\kappa ,\kappa_{+})=\left\{\begin{array}{cc} 0 & x<\kappa_{-}\\ p_{+}{\left(x-\kappa_{-}\right)}^{2}+r_{+}{\left(x-\kappa_{-}\right)}^{3} & \kappa_{-}<x<\kappa_{+}\\ x-\kappa & x\geq \kappa_{+} \end{array}\right.$$

(21) $$B(x|s=-1,\kappa_{-},\kappa ,\kappa_{+})=\left\{\begin{array}{cc} -(x-\kappa ) & x<\kappa_{-}\\ p_{-}{\left(x-\kappa_{+}\right)}^{2}+r_{-}{\left(x-\kappa_{+}\right)}^{3} & \kappa_{-}<x<\kappa_{+}\\ 0 & x\geq \kappa_{+} \end{array}\right.$$

for x is a scalar, $\kappa_{-}<\kappa <\kappa_{+}$ and

(22) $$\begin{array}{c} p_{+}=(2\kappa_{+}+\kappa_{-}-3\kappa )/{\left(\kappa_{+}-\kappa_{-}\right)}^{2}\\ r_{+}=(2\kappa +\kappa_{+}-\kappa_{-})/{\left(\kappa_{+}-\kappa_{-}\right)}^{3}\\ p_{-}=(3\kappa -2\kappa_{-}-\kappa_{+})/{\left(\kappa_{-}-\kappa_{+}\right)}^{2}\\ r_{-}=(\kappa_{-}+\kappa_{+}-2\kappa )/{\left(\kappa_{-}-\kappa_{+}\right)}^{3} \end{array}$$

The shape parameters $\kappa_{-}$, $\kappa_{+}$, and κ represent the lower side knot, the upper side knot, and the central knot, respectively. The first two knots define the change point between the functions, while the last influences the cubic and the linear functions. The ARESLab procedure automatically estimates the slope parameter and the number of Basis Functions to build the model. In particular, the design does not necessarily use both “complementary” basis functions; only the positive one or the negative one could be used in the estimation model.

In this study, the MARS algorithm for each sensor model in (1) is applied separately using the same data segment. Once the MARS spline basis functions are identified for all the monitored sensors, the local fault sensitivity matrix $\bar{W}(k)$ is analytically computed following the linearization procedure described in Section 2.

NOTE-2: Since there are no iterations (multiplications) between the sensor measurements, the fault diagnosis method based on the MARS algorithm could be implemented exclusively through the matrix product between suitably defined matrices and the values measured by the sensors. In particular, combining Equations (1), (3), and (19)–(22), it is possible to calculate the residual $r(k)=\Gamma (\cdots )\chi_{1-3}(k)+H(\cdots )\mu (k)$ where $\Gamma (\cdots )\in {\mathbb{R}}^{n_{x}\times \left(3n_{x}+1\right)}$, $H(\cdots )\in {\mathbb{R}}^{n_{x}\times \left(3n_{u}+1\right)}$, $\chi_{1-3}(k)=\left[\begin{array}{cccc} x+F & {\left(x+F\right)}^{2} & {\left(x+F\right)}^{3} & 1 \end{array}\right]\in {\mathbb{R}}^{3n_{x}+1}$, and $\mu (k)=\left[\begin{array}{cccc} u & u^{2} & u^{3} & 1 \end{array}\right]\in {\mathbb{R}}^{3n_{u}+1}$. Similarly, the matrix $\bar{W}(k)$ can also be calculated through the matrix product between some matrices appropriately defined and the values measured by the sensors. In particular, the column vector ${\bar{W}}_{j}(k)=\Omega (\cdots )\chi_{1-2}(k)$ where $\Omega (\cdots )\in {\mathbb{R}}^{n_{x}\times \left(2n_{x}+1\right)}$ and $\chi_{1-2}(k)=\left[\begin{array}{ccc} x+F & {\left(x+F\right)}^{2} & 1 \end{array}\right]\in {\mathbb{R}}^{2n_{x}+1}$. The matrices Γ(⋯), H(⋯) and Ω(⋯) depend on the shape parameters $\kappa_{-}$, $\kappa_{+}$ of each basis function of the model and the values of the sensor measurements. Therefore, the implementation complexity of the proposed approach is related to the matrix multiplication algorithm that results, as known, in the worst case $O(n^{3})$.

## 5. Machine Learning-Based Fault Isolation and Estimation

The proposed directional residual-based FI and FE scheme can be compared with machine learning (ML) solutions. ML techniques are extensively applied to FD problems; an extensive literature exists on this issue [32,33]. In this study, ML structures with different complexity are built and compared using the same data used to identify and test the directional residual-based technique. Specifically, FI and FE are addressed separately. First, a ML classifier is used to estimate the faulty sensor index. Then, a second ML approximator is used to estimate the fault amplitude. The first is a typical classification problem, while the second is a typical regression problem. Classification and regression structures are built by exploiting the dedicated MATLAB toolboxes in more detail. For example, support vector machines (SVMs) [17,22], neural networks (NNs) [23,24], decision trees [17,24], and ensemble of decision trees [25] structures have been considered.

### 5.1. Dataset Preparation for ML Algorithms

In contrast to the primary residual-based FI techniques that are based only on fault-free data and on fault directions, the ML technique requires faulty data samples which are representative of each possible sensor fault for a wide range of fault amplitudes.

Since real sensor flight data with sensor faults are not easily available, additive faults are artificially injected on the fault-free data to simulate the occurrence of a sensor fault (this approach of generating artificial faulty data is widely used in the FDi community, see for instance [11,12,13,14,15,16]).

For this reason, a new ‘ad-hoc’ data set is produced based on the fault-free dataset used to identify the MARS models. This is performed by adding a random amplitude fault at each sampling time on a randomly selected sensor. Random amplitude fault on a random sensor is clearly not a realistic fault scenario; this approach is used only to generate a rich set of training data to promote generalization capacity and robustness in the ML schemes.

It is observed that this simple fault generation method is possible thanks to the fact that, in this study, our approach is “memoryless”. Indeed, the estimation at the time k depends only on other signals at the same time k; therefore, serial temporal correlation of data has not been considered in the model. If the estimations at time k depend on signals at previous time instants (k−j), the above faulty data generation procedure can be easily extended by considering data segments of appropriate length. Next, the fault-free mean and standard deviation of the data are normalized, and two new labels (signals) are added to the data. The first is the index identifying the sensor where the fault is injected, while the second is the normalized amplitude of the fault.

### 5.2. ML Classifier for FI

The FI classifiers input is the vector $z_{FI}(k)=[x(k),u(k)]\in {\mathbb{R}}^{n_{x}+n_{u}}$ of the current sensors and inputs measurements, and the corresponding output is the label of the faulty sensors $S(k)=l\in [1,2\ldots n_{x}]$. The data set described in Section 6 is used for the training. The set of classifiers that are evaluated and the main design parameters are reported in Table 1.

### 5.3. ML Estimator for FE

The FE estimator input is the vector $z_{FE}(k)=[x(k),u(k),S(k)]\in {\mathbb{R}}^{n_{x}+n_{u}+1}$ of the current sensors and inputs measurements plus the index of the faulty sensor. The corresponding output is the amplitude of the normalized fault f(k)∈ℝ injected on the fault-free measurements. The data set described in Section 6 is used for the training. The set of estimators and the main design parameters are shown in Table 1.

### 5.4. Online Operation of ML Algorithms

**Figure 1:**
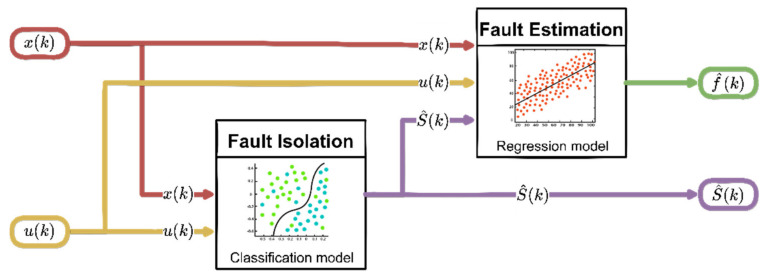
Online operation of the machine-learning-based FI and FE.

The previously trained ML structures are then used for FI and FE purposes in the online operation phase according to the scheme shown in Figure 1. Following the failure detection, the FI block processes the current input $z_{FI}(k)=[x(k),u(k)]$ and provides the estimation of the sensor fault index $\hat{S}(k)$ in the output. This information, as well as the measured current signals $z_{FE}(k)=[x(k),u(k),\hat{S}(k)]$, is then processed by the FE block that provides the estimation $\hat{f}(k)$ of fault amplitude at time k.

NOTE-3: The ML classification and regression schemes introduced in Section 5.2 and Section 5.3 have the same hyperparameters but differ in the inner architecture. For example, in the case of neural networks, the structure of the inner layers is the same, except the output layer, specifically in the case of a neural network classification, and the SoftMax activation function is applied. In contrast, linear activation functions are used in the regression neural network.

## 6. Semi-Autonomous Aircraft Flight Data

The FI and FE algorithms are designed and tested using flight data of a Tecnam P92 aircraft [34]. The data are acquired in a semi-autonomous mode; specifically, a pilot manually flew the plane during takeoff and approach/landing and flew autonomously at cruise conditions. A batch of six-flight datasets is considered in this study. The set of the 12 signals listed in Table 2 is considered [35], of which the first eight are the monitored sensors x(k), while the last four are the actuation (input) signals u(k). All the signals are normalized to zero mean and unitary variance.

Data from five flights (1 h and 21 min) are used to train models for a total of *N* = 48,661 data samples (with a data sampling period is 0.1 s). In contrast, the remaining batch of flight data (for a total of 11 min) is used for validation purposes for a total of N=6659 samples.

## 7. Design of the Non-Linear Directional Residuals

### 7.1. Redundancy Relation Identification

As described in Section 4, the MARS algorithm is used to identify the non-linear additive models introduced in Section 2. The MARS algorithm automatically selects the number of piecewise cubic splines and weights for each model in (1), exploiting a forward–backward iterative approach. Table 3 reports the model identification results for each of the additive models. The rows represent the identified model structure for each of the $n_{x}$ monitored sensors; the columns indicate the number of cubic splines selected by the algorithm to characterize the functions $g_{i,j}(x_{j})$ and $h_{i,j}(u_{j})$ in (1). It is observed that the algorithm does not choose all the available signals for modeling, thus also providing a feature selection.

Next, the same data are used to identify the linear redundancy relations parameters in (16) for each of the eight sensors. In this case, the model parameters are determined using standard least squares.

### 7.2. Accuracy of the Identified Additive Models

The first step to evaluate the effectiveness of the non-linear models is to compare their modeling accuracy with those of the linear models to estimate the sensor signals.

For each sensor, Table 4 reports the mean and standard deviation of the approximation error $e(k)=x(k)-\hat{x}(k)$ for the training and the test datasets in the form [*mean value ± standard deviation*]. The analysis of the table reveals that the non-linear models perform better than the linear ones. In fact, although the mean value of the estimation error for non-linear models is larger in some cases, this is still very close to zero. In contrast, the standard deviation of the estimation error is lower for non-linear models than linear models for all monitored sensors for both “Training” and “Test” data.

The higher accuracy provided by the non-linear additive models is considered important from an FI perspective because more accurate models can detect smaller amplitude faults by limiting the number of false alarms caused by modeling errors that could be equivocated with the occurrence of faults.

### 7.3. Fault Sensitivity Matrix

This section reviews in detail the time-dependent fault sensitivity matrix $\bar{W}(k)$ to highlight the relevant difference between linear and non-linear FI schemes. For this purpose, the non-linear models generated by the MARS algorithm are linearized following the approach proposed in Section 2 to build the matrix of fault signatures (i.e., the fault sensitivity matrix).

**Figure 2:**
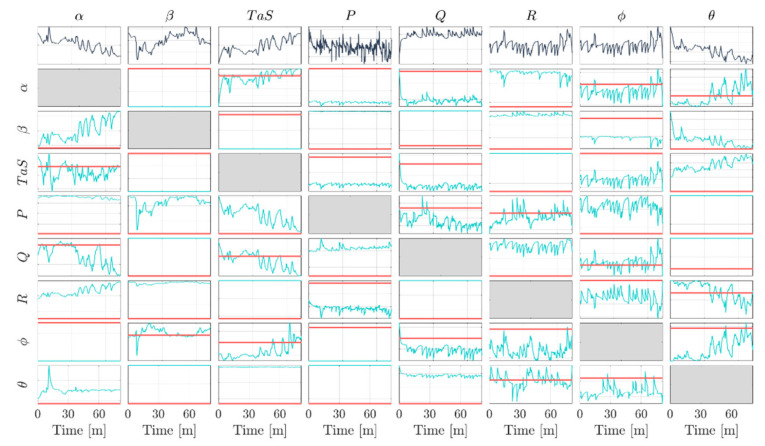
The first row reports the measured readings for the 8 monitored sensors. The cyan signals show the evolution of the (8 × 8) fault sensitivity matrix elements, displayed in Section 2. The red lines show the constant value of the elements of the fault sensitivity matrix W, shown in Section 2.1.

In Figure 2, the time evolution of the local fault sensitivities produced by the training data under no-fault conditions for a time segment of 1 h and 21 min is shown. A blank cell in the figure means that the MARS algorithm has not selected that signal in the corresponding additive model. The first row shows the monitored sensor readings [α,…,θ], while the other rows in the figure represent the time-dependent sensor fault direction at time k (cyan). In other words, the j-th column represents the evolution of the $n_{x}$ components of the vector ${\bar{w}}_{j}(k)$ (see Section 2). The figure also reports in red the fault sensitivities for the linear model case (fault directions) that are constant over time. In the last case, the j-th column represents the constant $n_{x}$ components of the vector $w_{j}$, as shown in Section 2.1. A detailed analysis of the data reveals that the time intervals where the local faults sensitivities change significantly are those in which the aircraft is executing a maneuver. The significant variation of the fault directions with time suggests a probable relevant effect on the FI performance compared to the linear case.

## 8. Design of Machine-Learning-Based FI Schemes

This section describes the design of ML-based FI schemes. First, the FI classifiers are trained. The input of the classifiers is the $z_{FI}(k)=[x(k),u(k)]\in {\mathbb{R}}^{12}$ vector of the eight monitored sensors and four inputs signals reported in Table 1. For each of the eight monitored sensors, single random amplitude faults are added to the fault-free signals at random time instants sampled from the training data. Positive and negative fault amplitudes are generated in the range $\left[\begin{array}{cc} -A_{\max} & A_{\max} \end{array}\right]$, whose values are reported in Table 5. The corresponding out at time *k* is the label of the faulty sensors S(k)=l∈[1,2…8]. A total of N=400,000 random samples are generated for the training. The training of the 16 FI classifiers (the first column in Table 6) is then performed using MATLAB. Table 6 shows the accuracy [36] of the models obtained from cross-validation of the training data with the n-fold validation taking n=5. The neural network FI classifiers achieved the highest accuracies compared to the other family of models; in particular, the wide neural network scores provided a 78.1%.

Next, the 16 FI estimators are trained. The input vector $z_{FE}(k)=[x(k),u(k),S(k)]\in {\mathbb{R}}^{13}$ coincides with $z_{FI}(k)$ augmented with the index S(k) indicating the faulty sensor, and the output is the amplitude of the normalized fault amplitude f(k)∈ℝ. A total of N=400,000 training samples are generated using the same procedure used for the training of the FI classifiers. The second column of Table 6 reports the root mean square error (RMSE), $RMSE=\sqrt{\frac{1}{N}{\sum_{i=1}^{N}\left(f_{i}-{\hat{f}}_{i}\right)}^{2}}$, of the models achieved, cross-validating the training data with the n-fold method (n=5). Once again, the wide neural network provides the lowest RMSE compared to the other family of models.

## 9. Metrics for Validating Fault Diagnosis Schemes

For validation and comparison purposes, additive constant bias faults of amplitude A(f(k)=A) are considered. The constant fault is applied at time k=1 and is maintained for the entire duration of the validation flight. The following FI and FE metrics are used.

### 9.1. Fault Isolation Percentage (FIP)

The FI performance is measured in terms of the fault isolation percentage (FIP), defined as:

${FIP}_{X}(A)$: Given a fault of amplitude A on the sensor X, the fault isolation percentage is the percent ratio between the number of samples the FI block which correctly isolates the faulty sensor and the number of samples in the validation flight.

(23) $$FIP=\frac{\#\;number\;of\;correctly\;isolated\;samples}{\#\;number\;of\;faulty\;samples}$$

The index $FIP_{X}(A)$ is calculated for each considered technique, for different fault amplitude A and for each monitored sensor X. The rest of the paper $FIP_{X}$ will refer to the average of $FIP_{X}(A)$ for the considered fault amplitudes injected on the monitored sensor X, i.e., $FIP_{X}=mean[FIP_{X}(A)]$. Similarly, $\bar{FIP}$ will refer to the average of the $FIP_{X}$ values evaluated over the eight monitored sensors, that is: $\bar{FIP}=mean[FIP_{X}]$.

### 9.2. Fault Estimation Percentage (FEP)

The primary residuals in models (1) and (16) for the non-linear and linear models, respectively, allow direct estimation (see Equation (15)) of the fault amplitude $\hat{A}$ that is computed as the difference between the measured and predicted signal. In contrast, the ML models estimate the fault amplitude $\hat{A}$ as a regression problem (see Section 5.3). The fault estimation percentage (FEP) is defined as:

$FEP_{X}(A)$: Given a fault amplitude A, the fault estimation percentage ratio is the absolute value of the percent ratio between the fault amplitude reconstruction. The $FEP_{X}(A)$ is calculated as the difference between the actual fault amplitude A and the mean of the reconstructed fault amplitude throughout the validation flight.

(24) $$FEP_{X}(A)=\left|\frac{A-\frac{1}{N}\sum_{i=1}^{N}{\hat{A}}_{i}}{A}\right|$$

Furthermore, the index $FEP_{X}(A)$ is calculated for each technique, a different fault amplitude A, and a monitored sensor X. In addition, $FEP_{X}$ refers to the average of $FEP_{X}(A)$ for the different fault amplitudes injected on the monitored sensor X, while $\bar{FEP}$ refers to the average of $FEP_{X}$ evaluate over the eight monitored sensors.

### 9.3. Complementary Fault Estimation Percentage (cFEP)

In order to achieve a performance metric that is 100% when the FE performance is perfect and 0% when it is completely unsatisfactory, the complementary *FEP* (*cFEP*) is defined as:

(25) cFEP=max(0,100−FEP)

From the cFEP index, the $cFEP_{X}(A)$, $cFEP_{X}$, and $\bar{cFEP}$ indices are derived. In addition, to produce an overall performance ranking that takes into account both the FI and FE performance, the overall performance index $J_{tot}$ is defined:

(26) $$J_{tot}=\frac{\bar{FIP}+\bar{cFEP}}{2}$$

Perfect performance is archived when $J_{tot}=100$, i.e., in the case of perfect fault isolation and perfect fault reconstruction.

## 10. Comparison between Directional Residual and Machine Learning Techniques

This section compares the fault diagnosis performance provided by the directional residuals and machine-learning-based methods. This study is performed using the data of the validation flight. Positive and negative constant faults are added to the fault-free data, considering, for each sensor, fault amplitudes *A* equal to ±17%, ±33%, ±50%, ±67%, ±83%, and ±100% of the maximum fault amplitude taken from Table 5. The faults are added at time k=1 to the faulty sensor and maintained constant for the entire flight duration. This procedure is repeated for all eight sensors and all the considered fault amplitudes, resulting in a total of about N=640,000 validation samples. The mean performance for all the sensors and fault amplitudes is evaluated using $\bar{FIP}$, $\bar{FEP}$, and $\bar{cFEP}$ indices already in Section 9. The results are reported in the first two columns of Table 7.

It is observed that the non-Linear technique (NL-DR) provides 71% in terms of $\bar{FIP}$. Although satisfactory, it is also observed that medium neural network (M-NN) and the wide neural network (W-NN) methods perform slightly better. On the other side, considering the $\bar{FEP}$ performance, the NL-DR achieves an excellent 18% while M-NN and the W-NN provides a significant performance degradation equal to 35%.

These facts are relevant because the NL-DR provides a high-level FI performance while maintaining an excellent capability for fault reconstruction. This fact does not apply to any one of the 16 ML techniques that provide a $\bar{cFEP}$ performance lower than 72%.

In summary, the best resulting method in terms of FI performance is the W-NN, and the worst is the F-SVM. Considering the FE performance, the best $\bar{cFEP}$ is provided by our proposed NL-DR method, and the worst is the L-SVM.

### 10.1. Overall Performance Comparison

The fourth column of Table 7 reports the index $J_{tot}$ for all the techniques. It is now evident that the resulting method with the best overall performance index is given by the proposed NL-DR (77%), followed by 3-NN (71%) and M-NN (69%) and by 2-NN (69%).

The last column of Table 7 also shows the combined memory occupancy of the isolation and estimation models. The SVM models have the highest memory occupation, up to about 50 MB, while the most parsimonious architectures are those based on directional residuals and neural networks.

### 10.2. In-Depth Performance Comparison of the Best Techniques

**Figure 3:**
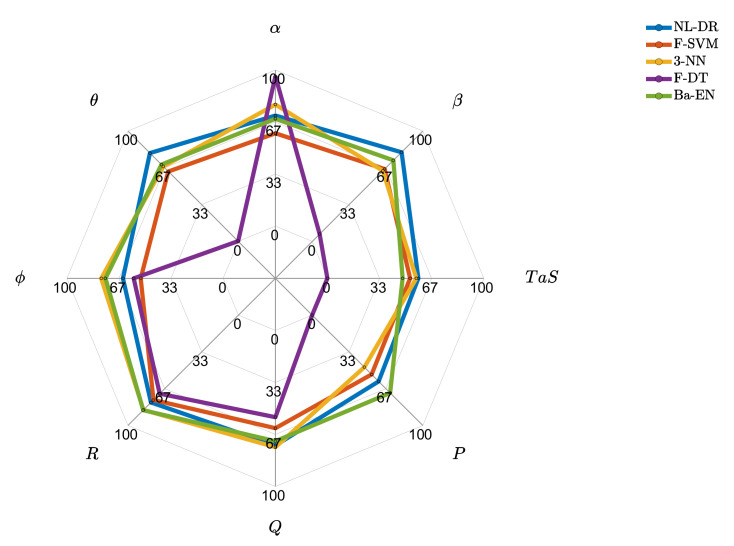
Fault isolation percentage for the best techniques for the 8 monitored sensors.

This section shows the $FIP_{X}$ and $cFEP_{X}$ indices for the best performing techniques explicitly evaluated for the eight monitored sensors. Figure 3 shows that the F-DT approach is biased toward the α sensor at the expense of the others. It perfectly isolates the faults on the α sensor, but it cannot correctly isolate any fault occurring on β, TaS, P, and θ sensors. It is also observed that the NL-DR technique performs better than the other techniques for four of the eight monitored sensors.

**Figure 4:**
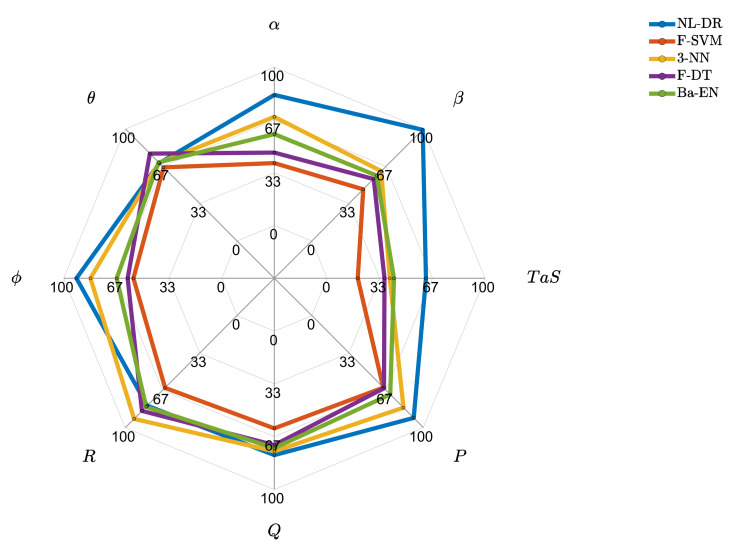
Fault estimation percentage for the best techniques for the 8 monitored sensors.

In Figure 4, it can be deduced that ML techniques have much lower FE performance than those provided by the NL-DR method for all the sensors. In fact, the proposed NL-DR scheme performs significantly better than the others for six of the eight monitored sensors.

### 10.3. Performance Comparison Evaluated over a Wider Fault Range

**Figure 5:**
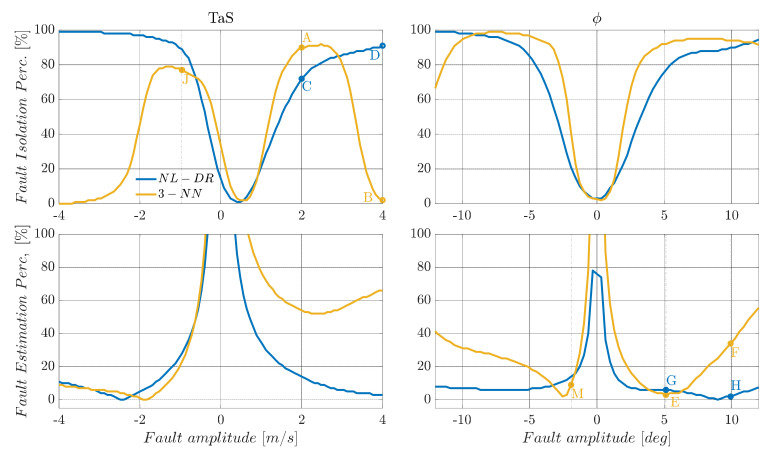
Fault diagnosis performance for *TaS* and *φ* sensors. The blue line is the NL-DR and the yellow line is the 3-NN trained with fault amplitude in the nominal ranges of Table 6.

The performance of any machine learning technique is strongly influenced by the data used for training. This implies that the available set training data strongly influences ML-based FIE methods in our specific case. In contrast, the proposed residual-based approach is virtually independent from the fault amplitude. In fact, in the primary residual modelling phase, no assumption is made about the magnitude of the faults, which leads to significant benefits in case the range of potential faults is under- or over-estimated. A clear example of this potential problem can be observed in Figure 5, where the response of $FIP_{X}(A)$ and $FEP_{X}(A)$ indices for TaS and ϕ sensors are compared for NL-DR and neural-network-based methods.

In the upper part of Figure 5, it can be observed that by injecting faults with amplitudes twice than the nominal ranges used for training (nominal range: $\left[\begin{array}{cc} -2 & 2 \end{array}\right]$ m/s for the TaS sensor and $[\begin{array}{cc} -6 & 6 \end{array}]{}^{\circ}$ for the ϕ sensor), the FI performance of the 3-NN deteriorates quickly with the increase in the fault amplitude.

Considering a failure on TaS equal to 2 m/s, the $FIP_{TaS}(2)$ index for the 3-NN is 90% (Point A), while a failure of 4 m/s the $FIP_{TaS}(4)$ descends to 2% (Point B). On the other hand, using the NL-DR technique, the $FIP_{TaS}(A)$ performance constantly increases with the fault amplitude (Points C and D), avoiding the paradox response provided by the ML methods. Similar considerations apply to the $FEP_{\phi }(A)$ index trend for faults on sensor ϕ.

In terms of FE performance, it is observed that both the TaS and ϕ the NL-DR approaches provide a suitable monotone decreasing trend for the $FEP_{X}(A)$ index with an increase in the failure amplitude. At the same time, there is a rapid degradation of the $FEP_{X}(A)$ performance outside the nominal training range in the NN case. Specifically, a constant 5° failure on ϕ produces a $FEP_{\phi }(5)$ of 3% (Point E), while a failure of 10° produces a $FEP_{\phi }(10)$ of 35% (Point F). Using the NL-DR approach, the $FEP_{\phi }(A)$ index increases from 6% for a failure of 5° (Point G) to 2% in the case of a failure of 10° (Point H). The above issue is a typical effect known as “Unseen Data Problem” or “generalization problem”. It refers to the inability to make reliable predictions in regions outside those explored in the training data.

In our study, a simple method to limit this important problem is to provide the ML algorithms with a broader range of fault amplitudes to cover the unexplored regions in the training phase. However, on the other side, the excessive widening of the fault ranges used in the training data can lead to the inability to discriminate accurately small faults.

**Figure 6:**
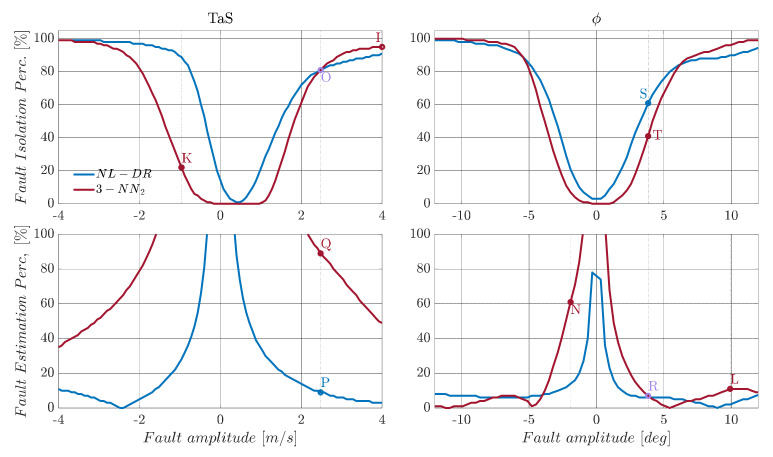
Fault diagnosis performance for *TaS* and *φ* sensors. The blue line is the NL-DR technique, and the amaranth line is the 3-NN_2_ trained in twice the fault range summarized in Table 6.

A direct example of this can be observed in Figure 6, where the tri-layer neural network is retrained (3-NN_2_), considering a range of fault amplitudes which are twice the size of the nominal range, as shown in Table 5. Using the retrained network, a relevant increase in the FI performance is achieved for large fault amplitudes, but at the expense of performance degradation for medium and small amplitude faults.

In more detail, consider the case of a failure of 4 m/s on TaS. In this case, the $FIP_{TaS}(4)$ provided by the retrained neural network (3-NN_2_) is now 95% (Point I), which is 93% more than the previous network (3-NN). Vice versa, in the case of a fault of −1 m/s, the 3-NN isolates the fault with an accuracy of 77% (Point J) while the 3-NN_2_ of 25% (Point K) confirms what is previously conjectured. Moreover, by analyzing the $FEP_{\phi }(A)$ index for the sensor ϕ, a similar conclusion can be drawn: that the $FEP_{\phi }(A)$ for 10° goes from 35% for 3-NN (Point F) to 9% for 3-NN_2_ (Point L), while a failure of −2° goes from 8% for 3-NN (Point M) to 60% for 3-NN_2_ (Point N), as shown in Figure 5 and Figure 6.

### 10.4. Overall Performance Evaluation for All the Monitored Sensors

To better compare the overall performance, Table 8, Table 9, Table 10, Table 11, Table 12, Table 13, Table 14 and Table 15 report, for each monitored sensor, the percentage ratio between the actual area below the $FIP_{X}(A)$ function (as those in Figure 5 and Figure 6) and the perfect performance area ($4A_{\max}\cdot 100\%$). The ideal area ratio is obviously 100%. The same process is also applied to the $cFEP_{X}(A)$ functions. The last column of the tables reports the mean between the $FIP_{X}(A)$ area ratio and the $cFEP_{X}(A)$ area ratio.

For almost all sensors, the area under the curve generated by 3-NN_2_ is larger than that under the curve generated by 3-NN, indicating a generalized performance improvement. However, in most cases, the performance improvement is only achieved for large amplitude faults, at the expense of performance degradation for small amplitude faults. This fact indicates the need to find a compromise on the magnitude of the faults used to train the neural network. This issue substantially limits the applicability of ML techniques, especially in a real-world context where an ‘a priori’ knowledge of the fault amplitude range cannot be established. Moreover, analyzing the results obtained for all the monitored sensors, the performance of the proposed NL-DR technique is almost always better than both the 3-NN and 3-NN_2_ techniques.

### 10.5. Time-Domain Performance Comparison with Same FIP/cFEP

This section evaluates and compares time domain FI and FE responses for the NL-DR and 3-NN_2_ techniques. In order to achieve a meaningful comparison, the tests are performed by selecting faults whose amplitudes are such that the two methods provide the same value for the $FIP_{X}(A)$ or the $cFEP_{X}(A)$ indices in Figure 5 and Figure 6, resulting in the selection of a fault of the amplitude of 2.5 m/s on TaS, and of 4° on ϕ, respectively (as expected, the faults are injected at k=1 and the constants for the whole flight are maintained).

**Figure 7:**
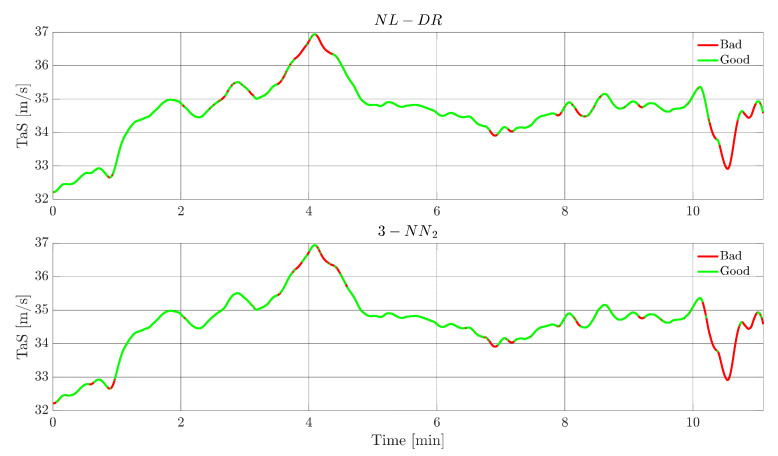
*TaS* sensor FI performance for a fault amplitude of 2.5 m/s in the test flight. The green segments indicate when the fault on the *TaS* sensor is correctly isolated by the FI scheme, while the red segments indicate a wrong FI.

Figure 7 shows, for a failure on *TaS*, the evolution of this signal. The green portions indicate the instants in which the FI is correct, while the red portions indicate when the failure is incorrectly attributed to a ‘wrong’ sensor. The upper plot refers to the NL-DR technique, while the lower plot refers to the 3-NN_2_ technique. For hypothesis, both methods isolate the fault with the same percentage (82%, Point O in Figure 6); the remarkable aspect is that the zones of wrong isolation are practically the same for the two techniques.

**Figure 8:**
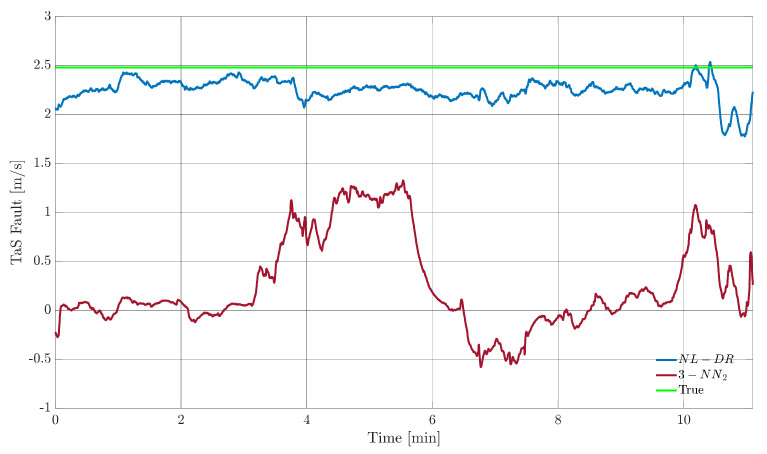
*TaS* sensor FE performance for a fault amplitude of 2.5 m/s (green line) during the test flight. The blue signal indicates the fault amplitude estimated by the NL-DR technique, while the amaranth signal indicates the fault amplitude calculated by the 3-NN_2_.

On the other side, there is a marked difference in $FEP_{X}(A)$ performance between the two techniques, see Figure 8. The fault amplitude estimated by the NL-DR method is much closer to the true value than the estimate provided by the 3-NN_2_ technique, as deduced from Figure 6 in Points P and Q, respectively.

**Figure 9:**
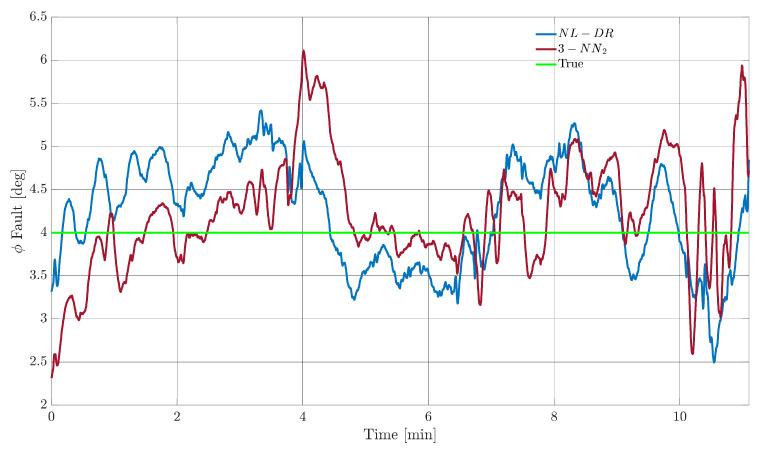
*φ* sensor FE performance for a fault amplitude of 4° (green line) during the test flight. The blue signal indicates the fault amplitude estimated by the NL-DR technique, while the amaranth signal indicates the fault amplitude provided by the 3-NN_2_.

**Figure 10:**
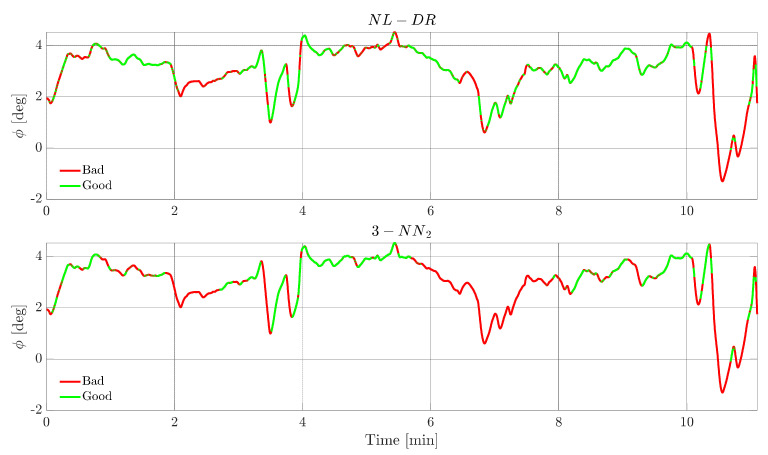
*φ* sensor FI performance for a fault amplitude of 4° during the test flight. The green segments indicate when the fault on the *φ* sensor is correctly isolated by the FI scheme, while the red segments indicate a wrong FI.

A similar analysis is then performed for the fault on the sensor ϕ. In this case, however, the fault amplitude is selected to be 4°, i.e., the point R of Figure 6 where the $FEP_{X}(A)$ index is equal to 6% for the two techniques. Figure 9 shows the evolution of the estimation of the fault amplitude for the two techniques, where it is confirmed that the performances are equivalent for all practical purposes. On the other side, from Figure 10, it can be observed that the $FIP_{X}(A)$ performance of the NL-DR technique is significantly better than that of the 3-NN_2_ technique (Points S and T in Figure 6).

## 11. Conclusions

The main purpose of the research effort described in this paper was to compare data-driven non-linear directional residual and machine-learning-based fault diagnosis techniques. The experimental study showed that the method based on primary residuals is virtually independent of the fault amplitude. Additionally, it was demonstrated that the FI and FE performance increases monotonically with increasing fault amplitude. In contrast, the performance of ML-based techniques depends heavily on the fault amplitudes used during training, producing potentially unpredictable results in regions not covered in the training phase. A partial solution to this problem was obtained by retraining the ML models using larger ranges for the faults injected in the training phase. The overall effect is that the FI and FE performance increases for large faults but, unfortunately, at the expense of a decrease in the estimation accuracy of small amplitude faults. In summary, it can be concluded that, while from the perspective of FI, the performance of residual-based and ML techniques is essentially equivalent, the residual-based approach results are more accurate and reliable than the ML-based approaches from the perspective of FE.

## Figures and Tables

**Table 1:** ML techniques for sensor fault isolation/estimation.

Name	Hyperparameters
**SVM**	
Linear SVM	kernel function: linear; kernel scale: automatic
Quadratic SVM	kernel function: quadratic; kernel scale: automatic
Cubic SVM	kernel function: cubic; kernel scale: automatic
Fine Gaussian SVM	kernel function: gaussian; kernel scale: 0.9
Medium Gaussian SVM	kernel function: gaussian; kernel scale: 3.6
Coarse Gaussian SVM	kernel function: gaussian; kernel scale: 14
**Neural Network**	
Narrow Neural Network	number of fully connected layers: 1, first layer size: 10
Medium Neural Network	number of fully connected layers: 1, first layer size: 25
Wide Neural Network	number of fully connected layers: 1, first layer size: 100
Bi-layer Neural Network	number of fully connected layers: 2, first layer size: 10; second layer size: 10.
Tri-layer Neural Network	number of fully connected layers: 3, first layer 10: second layer 10: third layer: 10
**Decision Tree**	
Fine Tree	maximum number of splits: 100
Medium Tree	maximum number of splits: 20
Coarse Tree	maximum number of splits: 4
**Ensemble Decision Tree**	
Bagged Trees	ensemble method: bag, maximum number of splits: 38935, number of learners: 30
Boosted Trees	ensemble method: AdaBoost, maximum number of splits: 20, number of learners: 30, learning rate: 0.1

**Table 2:** Aircraft signals.

*x*(*k*) (Monitored Sensors)				*u*(*k*) (Input Commands)	
*α*	Angle of attack	*P*	Roll speed	*Alt*	Altitude
*β*	Drifting angle	*Q*	Pitch speed	*Aie*	Aileron
*TaS*	True AirSpeed	*R*	Yaw speed	*Rud*	Rudder
*φ*	Roll angle	*θ*	Pitch angle	*Thr*	Thrust lever

**Table 3:** Additive model identification (the number of cubic splines selected by MARS algorithm).

	*α*	*β*	*TaS*	*P*	*Q*	*R*	*φ*	*θ*	*Alt*	*Aie*	*Rud*	*Thr*	*Tot.*
*α*		-	2	1	3	2	2	2	-	1	1	2	16
*β*	2		-	2	-	1	2	2	3	3	2	1	18
*TaS*	6	-		1	2	-	2	2	-	2	2	-	17
*P*	2	2	2		2	2	3	-	2	3	1	-	19
*Q*	3	-	2	2		2	5	-	1	-	-	2	17
*R*	1	1	-	2	-		2	1	3	2	3	2	17
*φ*	-	2	2	-	1	5		1	2	3	-	-	16
*θ*	2	-	2	-	1	3	3		-	-	2	4	17

**Table 4:** Sensors’ estimation accuracy [mean ± standard deviation].

	*α* [°]	*β* [°]	*TaS* [m/s]	*P* [°/s]	*Q* [°/s]	*R* [°/s]	*φ* [°]	*θ* [°]
L-Tr	−10^−14^ ± 1.1	10^−16^ ± 1.6	−10^−14^ ± 1.1	10^−14^ ± 1.5	10^−14^ ± 1.7	−10^−14^ ± 1.2	10^−15^ ± 1.2	−10^−14^ ± 1.4
N-Tr	10^−5^ ± 0.2	10^−4^ ± 0.5	−10^−4^ ± 0.2	−10^−3^ ± 0.6	10^−3^ ± 0.6	−10^−4^ ± 0.3	10^−4^ ± 0.3	10^−4^ ± 0.5
L-Te	−0.3 ± 0.2	−0.8 ± 0.7	−0.4 ± 0.4	10^−2^ ± 0.5	−10^−4^ ± 0.6	0.2 ± 0.4	0.1 ± 0.3	−0.1 ± 0.9
N-Te	−0.1 ± 0.1	−10^−2^ ± 0.5	−0.1 ± 0.1	10^−2^ ± 0.2	0.1 ± 0.2	−0.1 ± 0.2	−10^−2^ ± 0.1	−0.2 ± 0.3

(L: linear, N: non-linear; Tr: train data; Te: test data).

**Table 5:** Maximum fault amplitude.

	*α* [°]	*β* [°]	*TaS* [m/s]	*P* [°/s]	*Q* [°/s]	*R* [°/s]	*φ* [°]	*θ* [°]
$A_{\max}$	2	3	2	3	2	6	6	4

**Table 6:** Fault isolation and fault estimation performance evaluated on training data.

Name	Accuracy [%] {Fault Isolation}	RMSE {Faut Estimation}
**SVM**		
Linear SVM	34.1	0.77484
Quadratic SVM	67.5	0.7015
Cubic SVM	68.1	0.59263
Fine Gaussian SVM	59	0.58138
Medium Gaussian SVM	58	0.51841
Coarse Gaussian SVM	45.2	0.6992
**Neural Network**		
Narrow Neural Network	67.9	0.4875
Medium Neural Network	75.4	0.40438
Wide Neural Network	78.1	0.38077
Bi-layer Neural Network	71.2	0.41589
Tri-layer Neural Network	71.5	0.39589
**Decision Tree**		
Fine Tree	48.7	0.58246
Medium Tree	45	0.56079
Coarse Tree	40.9	0.57909
**Ensemble Decision Tree**		
Bagged Trees	46.5	0.4737
Boosted Trees	42.5	0.66543

**Table 7:** Performance comparison between directional residual-based and machine-learning-based techniques.

		$\bar{FIP}\;[\%]$	$\bar{FEP}\;[\%]$	$\bar{cFEP}\;[\%]$	$J_{tot}\;[\%]$	Memory Occup. (KB)
**DR**	**Directional Residuals**					
Lin-DR	Linear	64	33	67	66	1
*NL-DR*	*Non-Linear*	*71*	*18*	*82*	*77*	*10*
**SVM**	**SVM**					
L-SVM	Linear SVM	25	88	12	19	8000
Q-SVM	Quadratic SVM	62	76	24	43	45,000
Cu-SVM	Cubic SVM	62	62	38	50	45,000
F-SVM	Fine Gaussian SVM	61	48	52	57	60,000
M-SVM	Medium Gaussian SVM	55	53	47	51	55 000
Co-SVM	Coarse Gaussian SVM	16	77	23	20	70 000
**NN**	**Neural Network**					
1-NN	Narrow Neural Network	61	47	53	57	25
M-NN	Medium Neural Network	73	35	65	69	40
W-NN	Wide Neural Network	76	42	58	67	100
2-NN	Bi-layer Neural Network	68	30	70	69	30
3-NN	Tri-layer Neural Network	69	28	72	71	40
**DT**	**Decision Tree**					
F-DT	Fine Tree	36	38	62	49	1500
M-DT	Medium Tree	31	34	66	49	600
C-DT	Coarse Tree	24	40	60	42	200
**EN**	**Ensemble Decision Tree**					
Ba-EN	Bagged Trees	70	35	65	68	100,000
Bo-EN	Boosted Trees	32	75	25	29	500

**Table 8:** *α* area ratio.

Name	FIP [%]	cFEP [%]	Mean
NL-DR	80	81	81
3-NN	85	67	76
3-NN_2_	85	57	71

**Table 9:** *β* area ratio.

Name	FIP [%]	cFEP [%]	Mean
NL-DR	88	99	94
3-NN	77	55	66
3-NN_2_	88	63	76

**Table 10:** *TaS* area ratio.

Name	FIP [%]	cFEP [%]	Mean
NL-DR	72	75	74
3-NN	45	55	50
3-NN_2_	56	19	38

**Table 11:** *P* area ratio.

Name	FIP [%]	cFEP [%]	Mean
NL-DR	75	91	83
3-NN	55	70	63
3-NN_2_	70	75	72

**Table 12:** *Q* area ratio.

Name	FIP [%]	cFEP [%]	Mean
NL-DR	83	81	82
3-NN	83	61	72
3-NN_2_	80	84	82

**Table 13:** *R* area ratio.

Name	FIP [%]	cFEP [%]	Mean
NL-DR	77	78	78
3-NN	89	76	82
3-NN_2_	94	87	90

**Table 14:** *φ* area ratio.

Name	FIP [%]	cFEP [%]	Mean
NL-DR	77	89	83
3-NN	70	64	67
3-NN_2_	73	84	79

**Table 15:** *θ* area ratio.

Name	FIP [%]	cFEP [%]	Mean
NL-DR	86	78	82
3-NN	80	61	70
3-NN_2_	83	77	80

## Abbreviations

The following abbreviations are used in this manuscript:

AR	Analytical redundancy
cFEP	Complementary fault estimation percentage
DD	Data-driven
FD	Fault detection
FDi	Fault diagnosis
FE	Fault estimation
FEP	Fault estimation percentage
FI	Fault isolation
FIP	Fault isolation percentage
GAM	Generalized additive models
MARS	Multivariate adaptive regression splines
ML	Machine learning
NL	Non-linear
NN	Neural network
ReLU	Rectified linear unit
RMSE	Root mean square error
SVM	Support vector machine
